# An unusual case of autoimmune pancreatitis presenting as pancreatic mass and obstructive jaundice: a case report and review of the literature

**DOI:** 10.1186/1752-1947-5-253

**Published:** 2011-06-29

**Authors:** Nephertiti Efeovbokhan, Ashima Makol, Reuben V Cuison, Rebecca M Minter, Veera-Pavan Kotaru, Barbara A Conley, Sreenivasa R Chandana

**Affiliations:** 1 Department of Medicine, College of Human Medicine, Michigan State University, East Lansing, MI USA; 2 Department of Pathology, Sparrow Hospital, Lansing, MI, USA; 3 Department of Surgery, University of Michigan, Ann Arbor, MI, USA; 4 Division of Hematology and Oncology & Department of Medicine, College of Human Medicine, Michigan State University, East Lansing, MI, USA; 5 Department of Hematology and Medical Oncology, West Michigan Cancer Center, Kalamazoo, MI, USA

## Abstract

**Background:**

Autoimmune pancreatitis is a rare chronic inflammatory pancreatic disease that is increasingly being diagnosed worldwide. As a result of overlap in clinical and radiological features, it is often misdiagnosed as pancreatic cancer. We report the case of a patient with autoimmune pancreatitis that was initially misdiagnosed as pancreatic cancer.

**Case presentation:**

A 31-year-old Caucasian man presented to our hospital with epigastric pain, jaundice and weight loss. His CA 19-9 level was elevated, and computed tomography and endoscopic ultrasound revealed a pancreatic head mass abutting the portal vein. Endoscopic retrograde cholangiopancreaticography showed narrowing of the biliary duct and poor visualization of the pancreatic duct. Fine-needle aspiration biopsy revealed atypical ductal epithelial cells, which raised clinical suspicion of adenocarcinoma. Because of the patient's unusual age for the onset of pancreatic cancer and the acuity of his symptoms, he was referred to a tertiary care center for further evaluation. His immunoglobulin G4 antibody level was 365 mg/dL, and repeat computed tomography showed features typical of autoimmune pancreatitis. The patient's symptoms resolved with corticosteroid therapy.

**Conclusion:**

Autoimmune pancreatitis is a rare disease with an excellent response to corticosteroid therapy. Its unique histological appearance and response to corticosteroid therapy can reduce unnecessary surgical procedures. A thorough evaluation by a multidisciplinary team is important in rendering the diagnosis of autoimmune pancreatitis.

## Background

Autoimmune pancreatitis (AIP), also known as lymphoplasmacytic sclerosing pancreatitis, is a rare disease of the pancreas that is part of a systemic fibro-inflammatory syndrome complex. It is characterized by multi-organ immunoglobulin G4 (IgG4)-rich lymphoplasmacytic infiltration. The pancreas, biliary tree, salivary glands, retroperitoneum, lymph nodes and kidneys can be involved in this systemic fibrotic condition [1]. Although first described in 1961, it was not until 1995 that this disease was named "autoimmune pancreatitis" [2,3].

While the exact prevalence of AIP is unknown, the estimated prevalence is between 4.6% and 6% in patients with chronic pancreatitis and 11% in patients undergoing pancreatic resection for suspected malignancy [4,5]. There is a 2:1 male predominance. The patient's age at presentation is variable, with ranges reported from 30 to 80 years, with presentation commonly occurring in the sixth decade of life. The clinical manifestations are protean. Symptoms include abdominal pain, obstructive jaundice, weight loss, steatorrhea and new-onset diabetes mellitus [1]. Extra-pancreatic manifestations may also occur as part of the systemic fibro-inflammatory syndrome. Biliary tract strictures occur in the majority of patients. Lacrimal and salivary gland fibrosclerosis, retroperitoneal fibrosis, hypothyroidism and hilar lymphadenopathy have also been noted in some patients [6]. Some cases of pancreatic cancer have been reported in association with AIP. Recently, Kamisawa *et al*. [7] reported K-*ras *mutations in the pancreatobiliary tissue of patients with AIP, suggesting that AIP could be a risk factor for pancreatobiliary malignancy.

The diagnosis of AIP is challenging, as this disorder closely mimics pancreatic cancer [1]. We present the case of a man with obstructive jaundice, pancreatic head mass and elevated cancer biomarkers whose initial biopsy raised concern for pancreatic adenocarcinoma. His subsequent work-up revealed AIP as the etiology of his symptoms, and he was treated effectively with steroids, thus avoiding unnecessary surgery.

### Case presentation

A 31-year-old Caucasian man with no significant medical history presented to our hospital with a three-week history of epigastric pain radiating to his back. The pain was associated with non-bloody diarrhea and a 16-pound weight loss. His physical examination showed that he had scleral icterus with yellowish discoloration of his skin and mucous membranes. An abdominal examination revealed epigastric tenderness without rebound. Laboratory investigations revealed hemoglobin 13.9 g/dL, white blood cell count 10.6/μL, serum lipase 109 U/L, serum amylase 10 U/L, total bilirubin 10.6 mg/dl (direct and indirect fractions 8 mg/dL and 2.6 mg/dL, respectively). His fasting blood glucose level was elevated (> 250 mg/dL) in multiple readings. His liver enzymes were elevated (aspartate aminotransferase 110 U/L, alanine aminotransferase 231 U/L and alkaline phosphatase 589 U/L). Tests for hepatitis A, B and C were negative. The tumor marker CA 19-9 level was elevated at 3282 U/mL.

**Figure 1:**
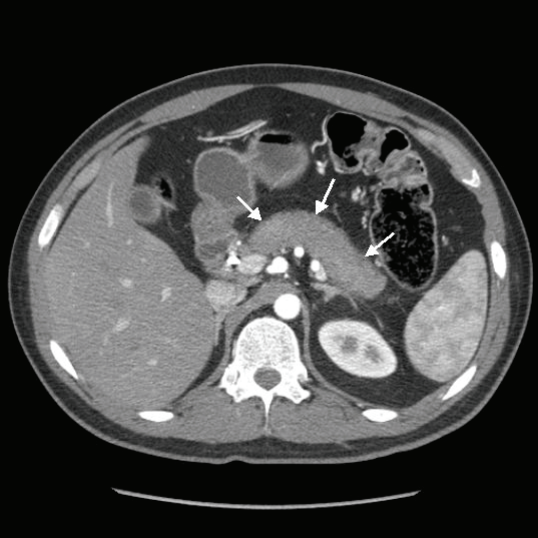
**Computed tomography of the abdomen showing homogeneous parenchymal enhancement with an almost "feathery" border and "sausage" appearance of the pancreas**.

Computed tomography (CT) of the abdomen showed a mass in the pancreatic head measuring 3.7 cm×3.2 cm abutting the portal vein. This finding was also seen in the endoscopic ultrasound (EUS). A fine-needle aspiration biopsy done at the time of EUS showed atypical ductal epithelial cells, which raised our clinical suspicions of adenocarcinoma. Endoscopic retrograde cholangiopancreaticography (ERCP) revealed mild narrowing of the distal common bile duct with proximal dilation and poor visualization of the pancreatic duct. After performing sphincterotomy, we placed a biliary stent, which led to significant improvement in the patient's symptoms and bilirubin level. He was also started on insulin therapy for his newly diagnosed diabetes mellitus. A presumptive diagnosis of pancreatic cancer was made by the referring physician. However, upon review by the medical oncology department, and because of his age, general condition and lack of family history of pancreatic cancer, the patient was referred to a tertiary care center for further evaluation. A contrast-enhanced CT scan (Figure 1) of the abdomen was repeated and showed a sausage-shaped pancreas with delayed rim enhancement. His serum IgG4 level was elevated at 365 mg/dL. A flexible sigmoidoscopy revealed features suggestive of ulcerative colitis (UC).

On the basis of the clinical, laboratory and imaging findings, a definite diagnosis of AIP was made and the patient was started on prednisone 40 mg/day. After four weeks, his biliary stent was removed, as the narrowing of the biliary duct had resolved. The dose of prednisone was tapered and stopped after 12 weeks. His insulin therapy was also stopped, as his blood glucose level had returned to normal. The patient did not receive treatment for UC, as he was asymptomatic. He is currently doing well.

## Discussion

Our ability to recognize AIP and differentiate it from pancreatic adenocarcinoma is aided by the use of formal Mayo Clinic, Japanese and Korean diagnostic criteria [8-10]. The Mayo Clinic HISORt criteria comprise five cardinal features involving findings regarding histology, imaging, serology, other organ involvement and response to corticosteroid therapy. In AIP, the pancreas is diffusely enlarged. The predominant histologic feature is infiltration of the peri-ductal space with plasma cells and T lymphocytes. In addition to this infiltrate, acinar destruction, obliterative fibrosis involving the major and minor veins and storiform fibrosis of the pancreatic parenchyma are present. The fibrosis may extend from the pancreas to contiguous peri-pancreatic soft tissue.

Various imaging modalities, including abdominal ultrasonography, CT, ERCP, EUS, magnetic resonance imaging (MRI) and magnetic resonance cholangiopancreatography may be used in the diagnosis of AIP. On CT scans, a diffusely enlarged pancreas with the appearance of a sausage-shaped organ is a classic marker for AIP. Delayed contrast enhancement, a rim-like capsule surrounding the gland on contrast-enhanced sequences (the hypoattenuation halo), a non-dilated, ectatic pancreatic duct and the absence of peri-pancreatic fat hypoenhancement are other common features seen on CT scans (reviewed in [11]). A study using dual-phase CT to identify findings that aid in differentiating AIP from pancreatic adenocarcinoma showed that patients with AIP were more likely to have diffusely decreased enhancement of the pancreas, capsule-like rim, pancreatic strands, pancreatic calcifications, bile duct wall enhancement and renal involvement [12]. MRI reveals enlargement of the pancreas with decreased signal intensity on T1-weighted images and increased signal intensity on T2-weighted images [11]. The most common finding on EUS scans is diffuse or focal pancreatic enlargement along with a diffusely hypoechoic parenchyma [11].

A variety of serum markers have been used in the differentiation of AIP from pancreatic carcinoma. Serum IgG4 level is considered one of the most sensitive and specific markers for AIP. A serum IgG4 level greater than 140 mg/dL was reported to have a sensitivity, specificity and positive predictive value of 76%, 93% and 36%, respectively, and at levels greater than 280 mg/dL, these values are 53%, 99% and 75%, respectively [13]. Our patient's IgG4 level was elevated at 385 mg/dL. The tumor marker CA 19-9 is another useful blood test in differentiating benign from malignant pancreatic disorders. The median sensitivity of CA 19-9 for detecting pancreatic adenocarcinoma is 79% (inter-quartile range (IQR), 70% to 90% and the median specificity is 82% (IQR, 68% to 91%) [14]. However, levels of CA 19-9 may also rise in some benign pancreatic conditions, including obstructive jaundice. The CA 19-9 level usually decreases after decompression of the biliary system. Our patient's CA 19-9 level was initially 3282 U/mL and decreased to 129 U/mL after biliary decompression. Recently, an anti-PBP peptide antibody was detected in 33 (94%) of 35 patients with AIP, compared with 5 (< 5%) of 110 patients with pancreatic cancer [15]. Other antibodies elevated in patients with AIP include anti-lactoferrin and anti-carbonic anhydrase II and anti-carbonic anhydrase IV, but these antibodies are available only for research purposes.

AIP has been shown to be responsive to corticosteroid therapy [11]. The decision to treat patients with a corticosteroid is most often based on symptoms, imaging features consistent with AIP, an elevated IgG4 level and a low suspicion of cancer. A histological diagnosis of AIP is usually not required. An EUS-guided core biopsy or laparoscopic biopsy can be obtained to rule out pancreatic cancer. The typical corticosteroid dose is prednisone 30 mg/day to 40 mg/day for four weeks, followed by slow tapering by 5 mg/week. Patients should be followed closely for clinical, radiological and serologic resolution, which may be seen as early as two to three weeks after the commencement of therapy. Recurrence is seen in up to 40% of patients, and these patients may benefit from another course of corticosteroids or from the use of immunosuppressive agents such as azathioprine [15]. In patients who do not respond to steroid therapy, the diagnosis of AIP should be re-evaluated.

## Conclusion

AIP is a unique chronic pancreatic disorder that occurs as part of a systemic fibro-inflammatory syndrome. Its diagnosis can be challenging, as it presents in a similar fashion to other pancreatic disorders, especially pancreatic adenocarcinoma. It has an excellent response to corticosteroid therapy. A thorough evaluation by experienced multi-disciplinary team is critical in diagnosing AIP, especially in young patients. Clinical suspicion of the entity is critical. Clinical experience and refinement of diagnostic criteria may help in the differentiation of this entity from pancreatic carcinoma.

## Consent

Written consent was obtained from the patient for publication of this case report and accompanying images. A copy of the written consent is available for review by the Editor-in-Chief of this journal.

## Competing interests

The authors declare that they have no competing interests.

## Authors' contributions

NE, AM and SC performed the literature search and wrote the manuscript. RVC was the pathologist who interpreted the pathology slides. RMM was the consultant gastroenterologist, acquired the images and critically reviewed the manuscript. BC and PK critically reviewed the manuscript. All authors read and approved the final manuscript.
